# Arrested Puberty in a Young Adult With a Macroprolactinoma: Case Report and Literature Review

**DOI:** 10.1155/crie/5388529

**Published:** 2025-01-31

**Authors:** Sánchez M., Otazú M., Furtenbach P., Piñeyro M.

**Affiliations:** Unidad Académica de Endocrinología y Metabolismo, Hospital de Clínicas, Facultad de Medicina, UdelaR, Montevideo, Uruguay

**Keywords:** delayed puberty, hyperprolactinemia, hypogonadism, hypogonadotropic, macroprolactinoma

## Abstract

Prolactinoma is the most common pituitary tumor, with clinical presentations varying according to sex, age of onset, tumor size, and prolactin (PRL) levels. These tumors are rare in the pediatric and adolescent populations. Hyperprolactinemia leads to hypogonadotropic hypogonadism, resulting in reproductive, metabolic, sexual, and skeletal consequences that can affect puberty development. Here, we present the case of a 23-year-old male patient diagnosed with arrested puberty secondary to a macroprolactinoma. The clinical presentation, diagnostic approach, therapeutic management, and a literature review are discussed.

## 1. Introduction

Prolactinomas are prolactin (PRL) secreting adenomas derived from lactotropic cells in the pituitary gland. They represent 40%–50% of pituitary adenomas and are the most frequent among functioning adenomas [1, 2]. The prevalence in adults is estimated at 100 cases per million people [3]. There is a 10:1 ratio between females and males, with the highest incidence occurring between 20 and 50 years old [3]. In the pediatric population, prolactinomas are rare, with an incidence of 0.1 per 100,000 inhabitants, representing less than 2% of all intracranial tumors [4]. However, diagnoses around puberty are on the rise [5]. Children and adolescents are more likely to have macroprolactinomas compared to adults.

Hyperprolactinemia leads to hypogonadism, which is associated with alterations in pubertal development, metabolic syndrome, and decreased bone mineral density (BMD) with an increased risk of fractures [6, 7].

We present a case report of a patient presenting visual field alteration and arrested puberty as clinical manifestations of a macroprolactinoma.

## 2. Case Report

A 23-year-old male patient with a personal history of obesity presents to the emergency room due to left temporal hemianopsia of 2 months duration. He reports a mild frontal headache that subsides with analgesics without associated nausea or vomiting. Additionally, he has poor facial hair development and decreased libido since adolescence. He denies muscle weakness and has not initiated sexual relations. There are no other signs of pituitary hypofunction.

**Figure 1:**
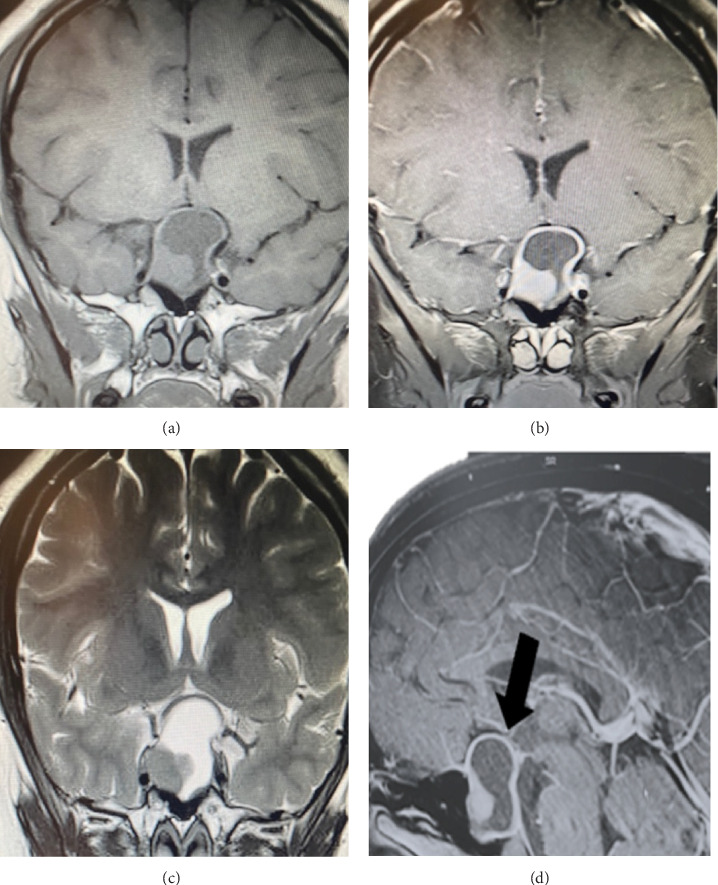
Pituitary MRI: (a) coronal T1-weighted, (b) coronal contrast-enhanced T1-weighted with gadolinium, (c) T2-weighted, and (d) sagittal T1-weighted. The images show a well-delimited, solid-cystic pituitary macroadenoma measuring 32 × 25 × 17 mm (black arrows). MRI, magnetic resonance imaging.

Pituitary magnetic resonance imaging (MRI) revealed a pituitary macroadenoma measuring 32 × 25 × 17 mm in its lateral (L), transverse (T), and anteroposterior (AP) diameters, respectively. The tumor was well-delineated, solid-cystic, and predominantly cystic (Figure 1). The patient was admitted with a presumptive diagnosis of pituitary adenoma.

Physical examination showed central-abdominal obesity with a BMI of 41.8 kg/m^2^ (weight 125 kg and height 173 cm). The patient was normotensive. His predicted height is 168 ± 10 cm. He had no facial hair. Confrontation visual field examination showed left temporal hemianopsia. There was no gynecomastia, and breast expression was negative. He did not have enlargement of hands or feet nor thickening of his facial features. No red vinous striae were observed. Axillary hair was present. Genital examination showed male genitalia, with a testicular volume of 4 cc, consistent with Tanner stage II (adult RV ≥ 15 ml). Pubic hair was Tanner stage III.

The hormonal assessment confirmed hyperprolactinemia, hypogonadotropic hypogonadism, secondary hypothyroidism, hypocortisolism, and hypovitaminosis D (Table 1). Computerized visual field testing reported left temporal hemianopsia and right superior temporal quadrantanopia.

With a diagnosis of macroprolactinoma with arrested puberty, cabergoline was initiated at increasing doses up to 2 mg/week, with good tolerance. The hormonal deficits were managed with oral hydrocortisone 15 mg/day by mouth (10 mg at 8:00 AM and 5 mg at 4:00 PM) and levothyroxine at 100 µg/day. Additionally, vitamin D supplementation at 5000 IU/day was started. The osteodensitometry test is pending.

**Figure 2:**
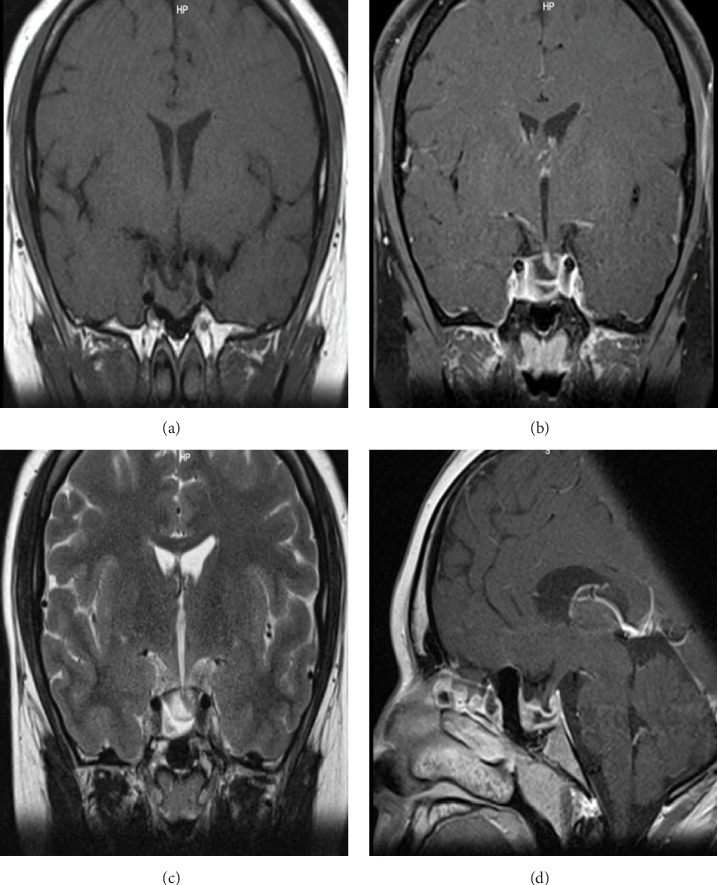
Pituitary MRI: (a) coronal T1-weighted, (b) coronal contrast-enhanced T1-weighted with gadolinium, (c) T2-weighted, and (d) sagittal T1-weighted. The images show a marked decrease in size of the known pituitary adenoma, measuring 10 mm (L) × 18 mm (T) × 18 mm (AP). The pituitary stalk is displaced laterally to the left. MRI, magnetic resonance imaging.

He noted an improvement in his visual fields and no longer experienced headaches. PRL levels decreased till 40 ng/ml, when the dose was increased to 2 mg/week. A pituitary MRI preformed 8 months after starting treatment revealed a marked decrease in size of the known pituitary adenoma, both in its solid and cystic components, now measuring 10 mm (L) × 18 mm (T) × 18 mm (AP). Currently, it does not occupy the suprasellar cistern nor compress the optic chiasm. The pituitary stalk is displaced laterally to the left (Figure 2).

Follow-up hormonal work-up showed increased cortisol levels. A standard high-dose ACTH stimulation test revealed recovery of the adrenal axis (cortisol: 19.8 µg/dl at 30 min and 20.9 µg/dl at 60 min), so hydrocortisone was discontinued. Levels of total testosterone (TT) and LH increased after start treatment with dopamine agonists, so hormone replacement therapy was not started.

Metabolically, he presents with grade III obesity without significant complications. He takes daily walks and has been evaluated by nutritionist, who recommended a hypocaloric diet plan.

## 3. Discussion

Prolactinomas constitute the most frequent subtype of pituitary adenomas in adolescents, followed by GH- and ACTH-producing adenomas [8]. At the time of diagnosis, boys more frequently present with macroadenomas (tumor ≥ 1 cm), while girls typically present with microadenomas (tumor < 1 cm) [9]. In the last 10 years, few cases of macroprolactinomas in adolescents presenting with delayed puberty have been published (Table 2). In relation to oncogenesis, germline mutations are more common in pediatric patients than in adults. Notably, mutations in the aryl-hydrocarbon receptor-interacting protein (AIP) gene have been identified in up to 20.5% of pediatric patients. Additionally, these tumors may be associated with multiple endocrine neoplasia type 1 (MEN 1) in up to 30% of cases [12–14]. The patient has no family history of endocrine pathology. Although the total calcium and albumin levels are normal, this does not completely rule out the possibility of primary hyperparathyroidism or MEN 1. Genetic testing was not performed.

In the pediatric population, the clinical manifestations of prolactinomas are primarily due to elevated PRL levels. These include delayed puberty (27% in boys vs. 48% in girls), galactorrhea (50% in boys vs. 30%–50% in girls), and gynecomastia in 60% of patients. In girls, primary or secondary amenorrhea occurs in 14%–40% and 30%–50% of cases, respectively. Additionally, neuro-ophthalmologic signs due to mass effects, such as visual disturbances and/or headaches, are more frequently observed at diagnosis in boys. Headaches are reported in 64%–77% in boys compared to 17%–30% in girls, and visual field disturbances are seen in 64% in boys compared to 7% in girls [15]. In a review of 77 macroprolactinomas in children and adolescents, the mean age of presentation was 16 years, and almost half of the cases presented with pubertal disturbances (49%) [11]. Hyperprolactinemia inhibits the hypothalamic–pituitary–gonadal axis in both sexes. This mechanism is proposed for hypogonadism rather than a mass effect in normal pituitary cells, as growth disturbance is seen less frequently than hypogonadism. For the diagnosis of hypogonadism in males, it is necessary to quantify serum levels of TT, which are expected to be decreased (less than 250 ng/dl), as seen in the case of this patient [16–18].

On the other hand, normal or low gonadotrophin (LH and FSH) levels establish the central origin of hypogonadism [19]. This disorder may delay the onset of puberty or appear during pubertal development, usually occurring after puberty has begun, as in this case, leading to arrested puberty [20].

Arrested puberty is defined as puberty that is not completed within 4–5 years from its onset to full gonadal development [21–23]. The first description of this phenomenon was by Howlett et al. [24].

The first sign of pubertal development in males is testicular growth (≥4 cc), which results from the development of the seminiferous tubules. This is followed by the appearance of pubic hair and later by penile growth [25]. The pubertal growth spurt begins at Tanner stage III, which coincides with the longitudinal growth of the penis [26]. Tanner stage II, as seen on physical examination, generally occurs at a mean age of 11.6 years (with a range between 9.5 and 13.7 years), at which time we suspect pubertal alteration began in this patient [26]. Furthermore, hypogonadism is closely related to metabolic syndrome and obesity [27, 28]. Low plasma testosterone levels are associated with obesity, as they lead to a decrease in muscle mass and an increase in visceral fat mass [29–32].

Other clinical manifestations include reproductive dysfunction (infertility, erectile dysfunction, azoospermia) and decreased BMD [20]. Hypogonadism leads to a progressive decrease and loss of BMD. This compromise is more significant during adolescence, as the peak of bone mass, which normally occurs around 25 years of age, is affected. The reversibility of this condition with the normalization of PRL levels is debatable, and therapeutic alternatives should be considered to prevent long-term skeletal complications [33]. Hypovitaminosis D is another factor that negatively affects BMD [34].

Treatment of hypogonadism should be based on the underlying cause. However, it may persist despite the reduction or eradication of the adenoma, necessitating androgen replacement therapy [35]. Evidence-based guidelines on optimal timing and regimen in puberty induction in males are lacking. Puberty induction is crucial to relieve psychological distress and enhance physical development. Testosterone therapy is the most common treatment due to its effectiveness and low cost. Treatment with gonadotropins like hCG and FSH promotes testicular growth and spermatogenesis in adolescent boys with congenital hypogonadotropic hypogonadism (CHH), unlike testosterone therapy. Common protocols involve using hCG alone or combined with FSH, with doses adjusted based on testosterone levels and clinical signs. Studies show significant testicular growth and sperm production with this treatment. Pretreatment with FSH optimizes future fertility, especially in those with complete gonadotropin deficiency and smaller testicular volume, who require both hCG and FSH for full testicular maturation [36].

In this patient, medical treatment with dopaminergic agonists (DA) is initiated; these are the first therapeutic options due to their efficacy, safety, and tolerance [37, 38].

The goals of therapy for macroprolactinoma are to reduce pituitary tumor mass, restore vision, ensure normal pubertal development, restore, and maintain adequate gonadal function, achieve optimal bone mass, and ensure future fertility [6, 37, 38]. Dopamine agonists are the first-line therapy, starting at low doses with individual dose modifications.

Regarding the adverse effects of DA, although their incidence in the pediatric population has not been systematically investigated, events similar to those observed in adults have been reported [39]. Remission has been demonstrated in 80%–90% of microprolactinomas and 70% of macroprolactinomas [9]. Despite treatment with high-dose cabergoline, drug resistance has been reported in 17.8% of macroprolactinomas and 10% of microprolactinomas [40]. Medical treatment should be maintained until the normalization of PRL concentrations and reduction of tumor size [2, 41]. Cystic prolactinomas may be resistant to volume reduction by DA. However, studies have shown significant shrinkage of cystic prolactinomas, including those with larger lesions that compress the optic chiasm [42, 43]. If there is no decrease in tumor size with DA, surgical treatment (transsphenoidal surgery) and, exceptionally, radiotherapy can be considered [10].

## 4. Conclusion

Health checkups during pubertal age are essential, as early detection of pubertal delay allows for prompt diagnosis and treatment. The management of pituitary adenomas requires a multidisciplinary approach. The first line of treatment of macroprolactinoma is medical therapy, which can effectively control the disease, restore PRL levels, and achieve normalization of the gonadal axis.

## Figures and Tables

**Table 1:** Laboratory.

Parameters	Before treatment	After 1 year of treatment	Reference values
	Result	Result	
Prolactin	1816	78.9	4–15.2 ng/ml
Cortisol 8 AM	5	—	6.2–19 µg/dl
TSH	2.80	1.66	0.27–4.2 ng/dl
T4L	0.69	1.07	0.93–1.7 ng/dl
Testosterone total	21.6	40.6	249–836 ng/dl
LH	<0.1	1.8	1.7–8.6 mUi/ml
FSH	<0.1	2.5	1.5–12.5 mUi/ml
IGF1	110	—	101–267 ng/ml
Serum calcium	9.2	—	8.5–10.5 mg/dl
Vitamin D	15	—	>30 ng/ml
Glycemia	90	83	70–100 mg/dl

Abbreviations: FSH, follicle-stimulating hormone; IGF1, insulin-like growth factor 1; LH, luteinizing hormone; T4L, free thyroxine; TSH, thyroid-stimulating hormone.

**Table 2:** Case reports.

Cases	Gender/age	Microprolactinoma, macroprolactinoma	Delayed puberty	PRL levels (ng/mL)	Treatment	Bibliography
1	M, 16	Macroprolactinoma	Yes	19,884	Cabergoline	[10]
2	M, 15			72,017		
3	M, 16			3428		
4	M, 14			930		
5	M, 15			202		[11]
6	M, 17			4200		
7	M, 18			278,000		[10]

Abbreviation: PRL, prolactin.

## Data Availability

The data supporting the findings of this study are available upon reasonable request from the corresponding author, Patricia Furtenbach (email: furtenbachpatricia@gmail.com).
